# Stimulation of synapse formation between stem cell-derived neurons and native brainstem auditory neurons

**DOI:** 10.1038/s41598-017-13764-8

**Published:** 2017-10-23

**Authors:** Zhengqing Hu, Zhenjie Liu, Xiaoyang Li, Xin Deng

**Affiliations:** Department of Otolaryngology-HNS Wayne State University School of Medicine Detroit, Michigan, 48201 USA

## Abstract

Integration of stem cell-derived cells into native cellular environment remains a challenge in the field. This study developed novel methods to co-culture neural stem cell-derived spiral ganglion-like neurons (ScNs) and mouse auditory cochlear nucleus (CN) neurons to understand whether ScNs of the peripheral nervous system (PNS) synapse with CN neurons of the central nervous system (CNS). ScNs were obtained from neural stem cells that were derived from transgenic mouse pre-labeled with enhanced green fluorescent protein (EGFP), whereas CN neurons were from postnatal mouse primary cultures. ScNs and CN neurons were co-cultured  for 4–6 days in the absence or presence of astrocyte-conditioned medium (ACM). Class III β-tubulin (TUJ1)-expressing connections were found between ScNs and CN neurons. Expression of the synaptic vesicle marker SV2 was significantly increased along connections between ScNs and CN neurons in the presence of ACM. Immunodepletion and knockout studies indicated that thrombospodin-1 played an important role in ACM-exerted synaptogenic effects. Newly-generated synapse-like structures expressed glutamatergic marker VGluT1, pre- and post-synaptic proteins. Synaptic vesicle recycling studies suggested functional synaptic vesicle retrieval. These results reveal that stem cell-derived PNS neurons are able to form functional connections with native CNS neurons, which is critical for stem cell-based neural pathway regeneration.

## Introduction

**Figure 1:**
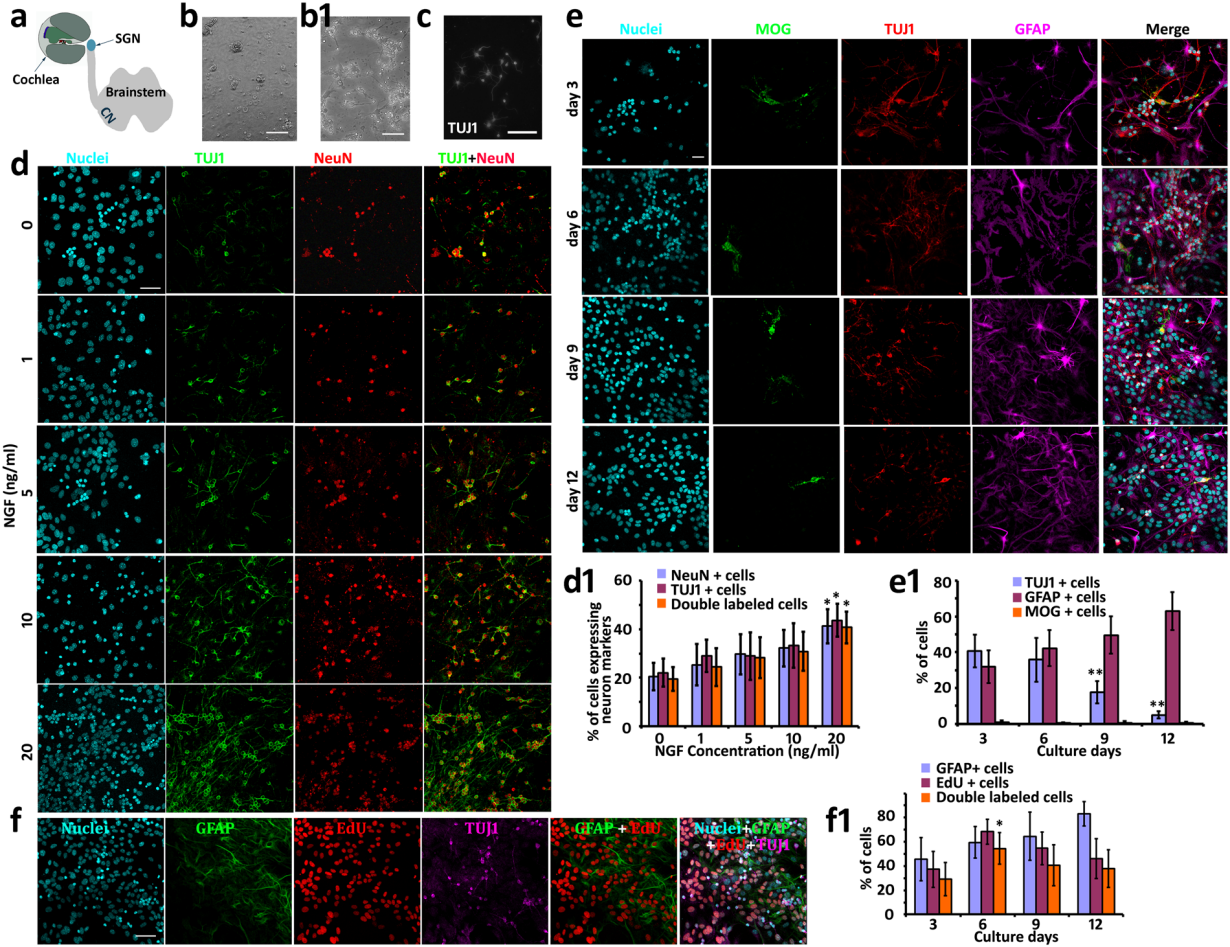
Dissociated cochlear nucleus (CN) neurons were maintained in culture for at least 12 days in the presence of nerve growth factor (NGF). (**a**) A diagram indicates the location of SGN and CN. (**b**) Singular cells and small cell clumps were observed after postnatal day 3 Swiss Webster mice CN tissues were dissociated. (**b1**) Dissociated CN singular cells and cell clumps adhered to the culture well after overnight culture. (**c**) Immunofluorescence showed TUJ1-expresssing cells after dissociated CN cells were cultured for 48 hr *in vitro*. (**d** and **d1**) A proportion of dissociated CN cells were double-labeled by neuronal markers TUJ1 and NeuN after 5 days in culture. The quantitative study revealed that the survival of CN neurons was promoted in the presence of NGF (mean ± standard deviation shown in the figure). Statistical analysis suggested that 20 ng/ml NGF exerted the best effects (**d1**), which was statistically significant (*indicates P < 0.05; ANOVA followed by Tukey post hoc test). (**e** and **e1**) Dissociated CN cells expressed the neuron protein TUJ1, glial cell protein GFAP, and oligodendrocyte protein MOG during culture days 3–12. The quantitative study indicated that TUJ1-expressing cells were observed for at least 12 days (mean ± standard deviation shown in the figure). The number of TUJ1-expressing cells decreased with the culture time, whereas the number of GFAP-expressing cells increased (**indicates P < 0.01; ANOVA followed by Tukey post hoc test). (**f** and **f1**) Dissociated CN cells, mainly GFAP-expressing cells incorporated EdU after exposure to EdU for 48 hr during culture day 1–3, 4–6, 7–9, and 10–12. Figure (**f**) showed typical images of EdU incorporation during culture day 4–6. In the quantitative study (**f1**), the number of GFAP-expressing cells increased with the culture time (mean ± standard deviation shown in the figure). However, EdU incorporation rates decreased after culture day 6, which may be due to the limited space in the culture well after 6 days’ culture (*indicates P < 0.05; ANOVA followed by Tukey post hoc test). Scale bar: 50 µm in (**a**–**f**).

Stem cell-based replacement has been suggested for regenerative medicine for a couple of decades. However, integration of stem cell-derived cells into the native cellular environment remains a largely understudied field. Auditory system regeneration was investigated in this research to address the synaptogenesis of stem cell-derived neurons. Spiral ganglion neurons (SGNs) convey sensory hair cell-perceived sound stimulations to cochlear nucleus (CN) neurons in the brainstem (Fig. 1a). However, SGNs are usually damaged in sensorineural hearing loss, which is a major disability affecting approximately 10% of the population. Neurotrophic factor supplement, stem cell-based replacement, and other approaches have been applied to treat SGN loss^1–6^. Pluripotent embryonic stem (ES) cells have been induced into cells expressing neuronal marker class III β-tubulin (TUJ1) and neurofilament^7–9^. Tissue-specific inner ear-derived multipotent neural stem cells (NSCs) have been generated and subsequently induced into cells expressing neuronal and SGN-like glutamatergic protein vesicular glutamate transporter 1 (VGluT1)^10,11^. These studies suggest that SGN-like neurons can be generated from pluripotent and multipotent stem cells. However, it remains unclear whether stem cell-derived neurons are able to integrate into the native auditory system, which at least includes neurite connections, myelination, and neural circuit function. This research focused on neurite connection between stem cell-derived SGN-like neurons and CN neurons, as this connection is critical for sound transfer from the ear to the brainstem.

The major obstacles of stem cell-based neurite connection include: (a) whether exogenous neurons are able to form synaptic connections with native CN neurons, and (b) how to stimulate stem cell-based synaptogenesis. Further, SGNs are peripheral nervous system (PNS) neurons, whereas CN neurons are central nervous system (CNS) neurons. It is unclear whether stem cells-derived PNS neurons are able to synapse with native CNS neurons. Additionally, the PNS-CNS connection of the auditory system resides within the bony temporal bone, which largely limits the access for *in vivo* research. To address these complicated issues, it is helpful to establish an *in vitro* stem cell-based co-culture model.

A novel co-culture system using stem cell-derived neurons and dissociated mouse CN neurons was developed in this study. In previous CN studies, the morphology and function of CN neurons have been investigated^12,13^. However, methods for maintaining CN neurons in the culture dish remain a largely understudied field. In this study, culture methods were developed to maintain mouse CN neurons *in vitro* for a novel co-culture system. Previously, NSCs were identified from wild type postnatal mouse SGN tissues, which were able to proliferate, express NSC proteins Sox2 and nestin, and differentiate into stem cell-derived spiral ganglion-like neurons (ScNs) expressing neuronal proteins including NeuroD, TUJ1, and neurofilament^10^. In the co-culture system developed in this research, ScNs were co-cultured with dissociated mouse CN neurons in order to understand whether ScNs are able to synapse with native CN neurons.

It has been demonstrated that neurotrophins including neurotrophin-3 (NT-3) and brain-derived neurotrophic factor (BDNF) are critical for SGN development and maintenance^14,15^. A recent *in vitro* report suggested that neurotrophins were able to stimulate neuronal differentiation and neurite extension of ScNs but not synapse formation^10^. Previous studies indicated that astrocyte conditioned medium (ACM) and thrombospodin-1 (TSP1) were able to stimulate synapse formation of CNS neurons *in vitro* ^16,17^, which was supported by a recent report in the application of ACM to ScN cultures^10^. It is noted that ACM and TSP1 stimulate synapse formation in a single type of neurons in these reports. It remains unclear whether ACM and TSP1 stimulate synaptogenesis between two types of neurons including synapse formation between PNS neurons and CNS neurons. In this research, we utilize our newly-developed co-culture system to determine whether ACM and TSP1 induce synaptogenesis between ScNs and CN neurons.

## Results

### Establishment of a postnatal mouse CN neuron primary culture system

Postnatal wild type mouse CN was dissected, followed by chemical and mechanical dissociation. Small cell clumps and singular cells were observed after dissociation (Fig. 1b), which adhered to culture wells after overnight culture (Fig. 1b1). Immunofluorescence showed that some dissociated CN cells expressed the neuronal protein TUJ1 after 48 hr in culture (Fig. 1c).

Because CN neurons were expected to co-culture with ScNs, nerve growth factor (NGF) was added to the culture medium to support the survival of CN neurons. Some CN cells expressed neuronal proteins TUJ1 and NeuN after they were cultured for 5 days (Fig. 1d). TUJ1 and NeuN double-labeled cells were approximately 19.46 ± 4.96% (mean ± standard deviation), 24.39 ± 7.89%, 28.25 ± 8.50%, 30.89 ± 8.13%, and 40.76 ± 6.54% in the cultures supplemented with 0, 1, 5, 10, and 20 ng/ml NGF respectively (Fig. 1d1). One-way analysis of variance (ANOVA) revealed that the 20 ng/ml NGF group showed the best neuron survival rate, which was statistically significant and thus selected for the following CN culture study (P < 0.05; Fig. 1d1).

To study the survival of neural cells including neurons, astrocytes, and oligodendrocytes, dissociated CN tissues were maintained in culture for 3, 6, 9, and 12 days in the presence of 20 ng/ml NGF. Immunofluorescence showed that the majority of CN cells expressed TUJ1 or GFAP, but small levels of myelin oligodendrocyte glycoprotein (MOG)-expressing cells were observed (Fig. 1e), indicating that dissociated CN tissues may contain neurons, astrocytes, and oligodendrocytes. The percentages of neurons and astrocytes at culture day 3 were 40.71 ± 8.99% (mean ± standard deviation) and 31.83 ± 9.06% respectively, which became 35.81 ± 12.16% and 42.30 ± 10.15% at culture day 6, and 17.75 ± 6.28% and 49.56 ± 10.48% at culture day 9. At culture day 12, 4.96 ± 1.95% neurons and 63.03 ± 10.71% astrocytes were still observed in culture (Fig. 1e1). It is noted that the percentage of neurons decreased with the culture time, whereas the percentage of astrocytes increased. To determine whether astrocytes proliferated in culture, EdU was added to the culture medium at culture day 1, 4, 7, 10 and maintained for 48 hr^10^. EdU incorporation study demonstrated that a number of GFAP-expressing astrocytes entered the S-phase during culture day 1–12 (Fig. 1f,f1), indicating that increased percentage of astrocytes was due to cell proliferation. None of TUJ1-expressing neurons were found to incorporate EdU, suggesting that neurons did not grow in culture. These studies indicate that CN neurons can be maintained in culture for at least 9–12 days, which is essential for developing a novel co-culture system to study synapse formation.

### Development of a novel stem cell-based ScN-CN co-culture system

**Figure 2:**
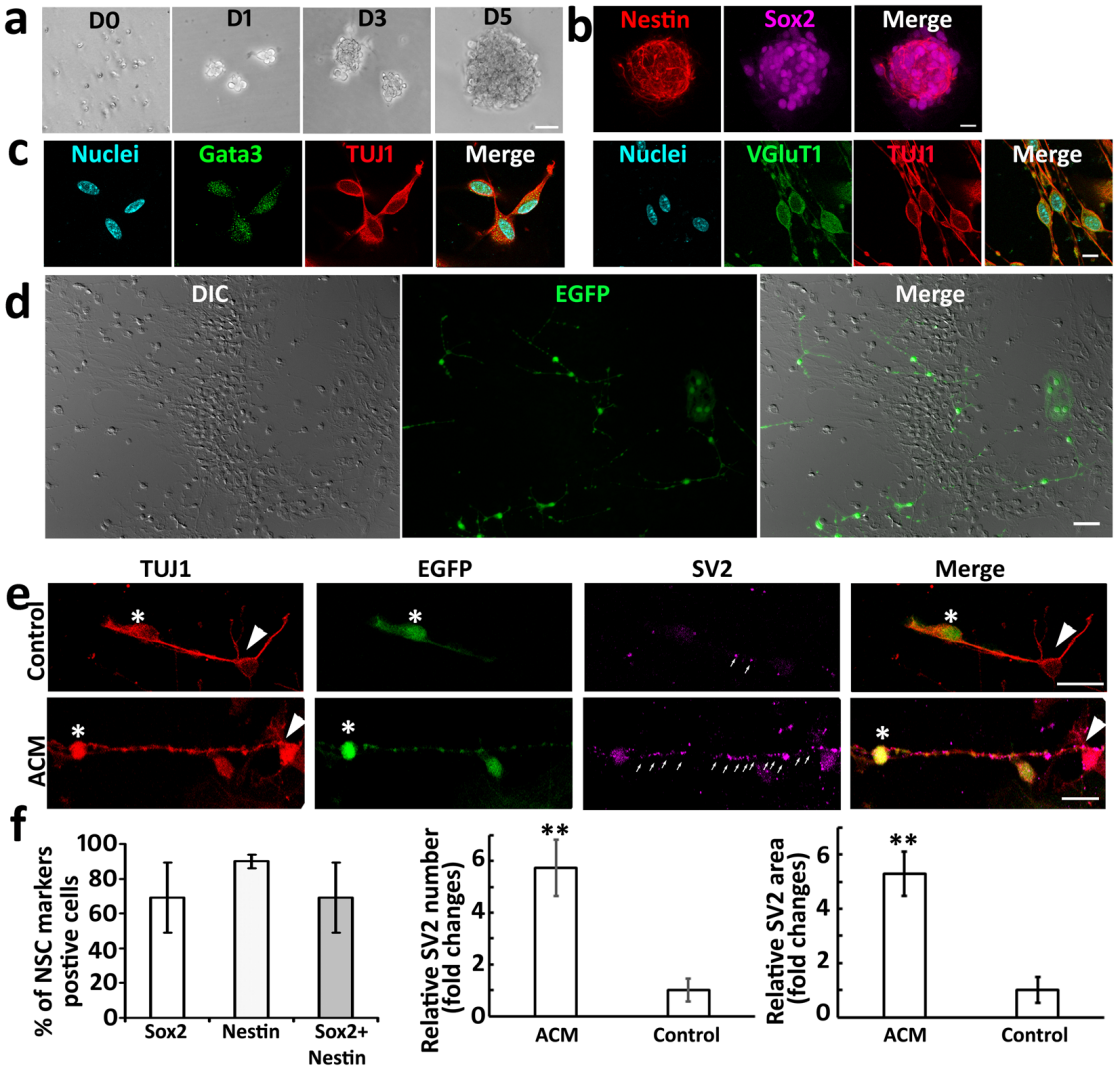
EGFP-ScNs formed connections with wild type mouse CN neurons. (**a**) Primary culture of postnatal day 3 EGFP-mouse SGN-derived cells formed spherical structures in suspension culture during culture day 0–5 (D0-D5). (**b**) Passage 3 EGFP-mouse SGN-derived neural spheres expressed NSC proteins Sox2 and nestin in suspension culture. (**c**) EGFP-mouse SGN-derived NSCs were induced into neurons (ScNs) expressing neuronal protein TUJ1, otocyst-derived cell protein Gata-3, and glutamatergic protein VGlut1 after seeding to the culture wells for 8–12 days. (**d**) EGFP-ScNs were co-cultured with dissociated wild type postnatal mouse CN cells for 5 days. EGFP indicated the cells originated from EGFP-mouse SGN-derived cells, whereas CN cells were observed by DIC microscopy without EGFP expression. (**e**) After co-culturing for 4–6 days, TUJ1 positive connections were observed between EGFP-ScNs (asterisks) and wild type CN neurons (arrowheads). In the control group (co-cultures without ACM supplement), a few SV2 positive puncta (arrows) were observed along connections between the ScN and CN neuron. In contrast, a number of SV2 puncta were found along connections in the ACM group (co-cultures supplied with ACM). (**f**) The quantitative study showed percentage of NSC proteins Sox2, nestin, and double-labeled cells in passage 3 SGN-derived neural spheres. The quantitative study showed that relative number and area of SV puncta per connection were significantly larger in the ACM group (mean ± standard error shown in the figure; **indicates P < 0.01, Mann-Whitney U test). Scale bar: 50 µm in (**a** and **d**), 25 µm in (**b** and **e**), and 10 µm in (**c**).

In this study, SGN tissues were obtained from transgenic mice expressing EGFP under control of the ubiquitous promoter CAG and suspended in the culture medium to identify NSCs using previously reported methods^10^. The neural spheres were formed in the primary and subsequent cultures for at least 5–8 weeks (Fig. 2a), which expressed NSC proteins Sox2 and nestin (Fig. 2b), indicating that these cells were NSCs. Passage 3–5 NSCs were induced into ScNs expressing EGFP and neuronal marker TUJ1 after 8–12 days in culture (Fig. 2c), which rendered sufficient time for the co-culture study. Additionally, ScNs expressed the otocyst-derived cell protein Gata3 and glutamatergic protein VGluT1 (Fig. 2c), indicating that they were SGN-like neurons. Therefore, EGFP-ScNs were used to co-culture with wild type CN neurons.

In the newly-developed co-culture system, dissociated wild type postnatal mouse CN tissues were cultured in the presence of NGF for 20–24 hr. After CN tissues and cells adhered, EGFP-ScNs were added to CN cultures. Both types of cells distributed in culture wells after overnight culture (Fig. 2d). After co-culturing for 4–6 days, immunofluorescence showed connections linking CN-CN neurons, ScNs-ScNs, and ScN-CN neurons, in which ScN-CN connections were focused in this study. TUJ1-expressing connections were found between ScNs and CN neurons, indicating that neurite connections were likely formed between these two types of neurons. However, synaptic vesicle immunostaining using anti-SV2 antibodies did not show significant SV2 immunostaining between ScNs and CN neurons (Fig. 2e, the control group), indicating that exogenous proteins and/or factors may be required to stimulate synaptogenesis between these two types of neurons.

### Stimulation of neuronal connections between ScNs and CN neurons by ACM

In the Bradford assay, the total protein concentration of concentrated ACM was 3,387 ± 247 µg/ml (mean ± standard deviation). In a 1:10 dilution, approximately 339 µg/ml ACM was added to the co-cultures. In this study, ACM was applied to the novel co-culture system to test whether ACM stimulated synaptogenesis between (a) two different types of neurons, (b) PNS neurons and CNS neurons, and (c) stem cell-derived ScN and native mouse CN neurons.

The synaptic vesicle protein SV2 immunostaining was used to evaluate the synaptogenic effect of ACM supplementation. Immunostaining showed that EGFP-ScNs and wild type CN neurons were labeled by anti-TUJ1 antibodies (Fig. 2e). Very few SV2 puncta were observed along connections between ScNs and CN neurons in the control group, whereas many SV2 puncta were found in the ACM group (co-cultures supplemented with ACM; Fig. 2e). The quantitative study of SV2 puncta along ScN-CN connections revealed that relative number and area of SV2 puncta in the ACM group were significantly larger than those of the control group without ACM supplement (Fig. 2f; P < 0.01, Mann-Whitney U test). These studies indicate that ACM likely stimulates expression of synaptic vesicle proteins along connections between ScNs and CN neurons.

### Identification of TSP1 as a crucial factor responsible for ACM-induced neural connections between ScNs and CN neurons

The new co-culture system was applied in this study to test whether TSP1 played an essential role in ACM-induced neural connection between ScNs and CN neurons. To determine the effect of TSP1, three sets of co-culture experiments were performed, which included evaluation of SV2 puncta in the presence of: (a) different concentration TSP1, (b) TSP1-immunodepleted ACM (TSP1-ID-ACM), and (c) ACM collected from TSP1-knockout mouse (TSP1-KO-ACM).

**Figure 3:**
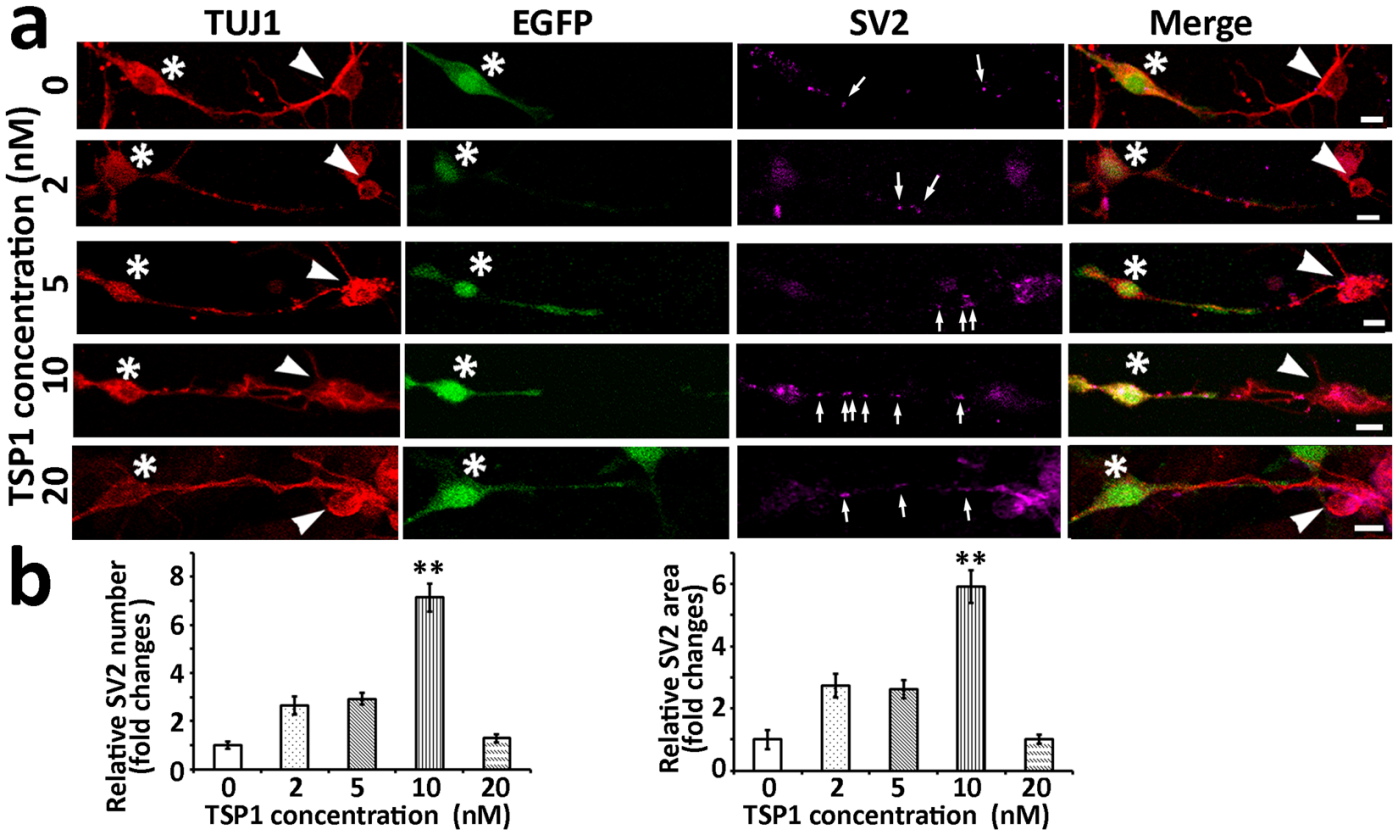
TSP1 stimulated expression of the synaptic protein SV2 along connections between EGFP-ScNs and CN neurons. (**a**) Immunofluorescence showed that TUJ1-expressing connections were found between EGFP-ScNs (asterisk) and wild type CN neurons (arrowhead). The number of SV2 puncta (arrows) along connections increased when 2–20 nM TSP1 was supplied. (**b**) The quantitative study suggests that the 10 nM TSP1 group exerted significant effects to the expression of SV2 puncta, including relative number and area of SV2 puncta (mean ± standard error shown in the figure; **indicates P < 0.01; ANOVA followed by Tukey post hoc test). Scale bar: 10 µm in (**a**).

TSP1 (0, 2, 5, 10, and 20 nM) was added to ScN-CN co-cultures and maintained for 4–6 days. Immunofluorescence showed that very few SV2-expressing puncta were observed along connections between ScNs and CN neurons in 0 (control), 2, 5, and 20 nM TSP1 groups, whereas many SV2-expressing puncta were found in the 10 nM groups (Fig. 3a). Statistical analysis indicated that the 10 nM group had significantly more SV2-expressing puncta (relative number and area of SV2 puncta) than other groups (P < 0.01, ANOVA; Fig. 3b); therefore, 10 nM TSP1 was selected for the following studies.

**Figure 4:**
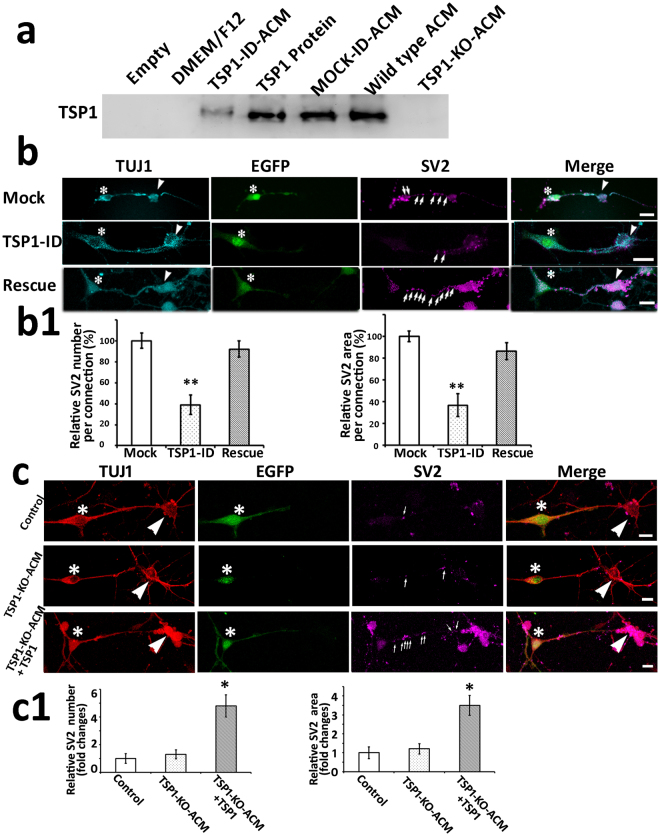
TSP1 immunodepletion or TSP1 knockout abolished ACM-exerted effects, which was rescued by the supplementation of exogenous TSP1 protein. (**a**) TSP1 antibodies were used for western blotting, which showed clear TSP1 bands in TSP1, mock, and ACM groups, a weak band in TSP1 immunodepleted ACM group (TSP1-ID-ACM), and no bands in empty, DMEM/F12, or TSP1 knockout ACM (TSP1-KO-ACM) groups. The image was cropped and inverted from a digital image captured by UVP imaging system (original images with ladder in the supplemental Fig. 1). (**b**) Immunofluorescence shows TUJ1-expressing connections between EGFP-ScNs (asterisks) and wild type CN neurons (arrowheads). A number of SV2-expressing puncta were observed along connections in the mock group (co-cultures supplied with the wild type ACM that has been immunodepleted by mouse IgG). The number of SV2-expressing puncta (arrows) along connections was reduced in the TSP1-ID-ACM group (co-cultures supplied with TSP1-ID-ACM), whereas it was increased when exogenous TSP1 protein (10 nM) was supplemented (the rescue group). (**b1**) The quantitative study indicates that the relative number and area of SV2 puncta decreased in the TSP1-ID-ACM group, whereas they significantly increased in the rescue group (mean ± standard error shown in the figure; **indicates P < 0.01; ANOVA followed by Tukey post hoc test). (**c**) Immunofluorescence showed TUJ1-expressing connections between EGFP-ScNs (asterisks) and wild type CN neurons (arrowheads). A few SV2-expressing puncta (arrows) were observed along connections in the control (co-cultures without ACM supplement) and TSP1-KO-ACM groups (ACM collected from TSP1 knockout mice). In contrast, many SV2-expressing puncta were found along connections in the rescue group (co-cultures supplies with TSP1-KO-ACM in the presence of exogenous 10 nM TSP1 protein).(**c1**) The quantitative study shows that relative number and area of SV2 puncta along connections were lower in the control and TSP1-KO-ACM groups, whereas they were significantly higher in the rescue group. Statistical analysis showed significant difference (mean ± standard error shown in the figure; *indicates P < 0.05; ANOVA followed by Tukey post hoc test). Scale bar: 20 µm in (b), and 10 µm in (**c**).

Dynabeads conjugated with antibodies specific for TSP1 were added to wild type ACM to obtain TSP1-ID-ACM, which showed weak TSP1 expression compared to the wild type ACM and mock-ID-ACM in western blotting (Fig. 4a and supplemental Fig. 1). Mock- and TSP1-ID-ACM were added to co-cultures for 4–6 days, and many SV2 puncta were observed in the mock-ID-ACM group, whereas very few SV2 puncta were found in the TSP1-ID-ACM group (Fig. 4b). In the rescue group, in which 10 nM TSP1 was added to TSP1-ID-ACM-treated co-cultures, significantly increased SV2 puncta (relative number and area) were detected along connections between ScNs and CN neurons (P < 0.01, ANOVA; Fig. 4b,b1). These studies revealed that TSP1 immunodepletion abolished ACM-induced effects, but addition of TSP1 protein was able to rescue it, suggesting that TSP1 may play a major role in ACM-exerted effects.

To further determine the role of TSP1, TSP1-KO-ACM was collected from TSP1-KO mice, which did not show TSP1 expression in western blotting (Fig. 4a). TSP1-KO-ACM was added to ScN-CN co-cultures for 4–6 days. Very few SV2 puncta were observed in the control and TSP1-KO-ACM groups, whereas many SV2 puncta were observed in the rescue group (TSP1-KO-ACM-treated co-cultures supplemented with 10 nM TSP1 protein; Fig. 4c). Quantitative study of SV2 puncta along ScN-CN connections revealed that relative SV2 puncta number and area were significantly higher in the rescue group (P < 0.05, ANOVA; Fig. 4c1). This study indicates that ACM-exerted effects were reduced in ScN-CN co-cultures treated with TSP1-KO-ACM, but can be rescued by the supplementation of purified TSP1 protein.

In the above three sets of studies, exogenous TSP1 exhibited concentration-related effects. TSP1 immunodepletion and knockout abolished ACM-induced synaptic vesicle protein expression, which was rescued by TSP1 protein supplementation. These studies suggest that TSP1 may play an important role in ACM-exerted effects in ScN-CN co-cultures.

### Characterization of neural connections between ScNs and CN neurons

**Figure 5:**
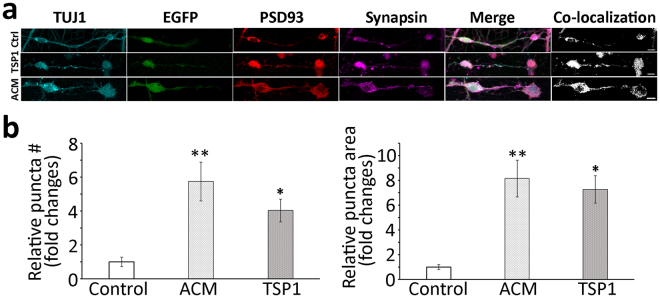
New connections between EGFP-ScNs and CN neurons expressed synaptic proteins. (**a**) TUJ1-positive connections were observed between EGFP-ScNs and wild type CN neurons, which also expressed presynaptic marker synapsin and postsynaptic marker PSD93 in the control (Ctrl; co-culture without supplement), TSP1, and ACM groups. Confocal microscopy-based co-localization showed obvious apposition of synapsin and PSD93 in the control, ACM and TSP1 groups (Person’s correlation 0.718 ± 0.057, Pearson’s R test, Leica LAS AF Lite Co-localization software). (**b**) The quantitative study showed that relative number and area of puncta along connections were lower in the control group, whereas they were significantly higher in the ACM and TSP1 groups (mean ± standard error shown in the figure; *indicates P < 0.05, **indicates P < 0.01, ANOVA followed by Tukey post hoc test). Scale bar: 10 µm in (**a**).

Antibodies specific for pre- and post-synaptic proteins were used to determine whether ACM- and TSP1-induced synapse-like structures expressed pre- and post-synapse proteins. It was found that synapsin and PSD93 were expressed in the control, ACM- and TSP1-induced synapse formation, which was further shown by confocal microscopy-based co-localization (Person’s correlation 0.718 ± 0.057, Pearson’s R test, Leica LAS AF Lite Co-localization software; Fig. 5). The quantitative study exhibited significantly more puncta along ScN-CN connections in the ACM and TSP1 groups (P < 0.01 for ACM group and P < 0.05 for TSP1 group, ANOVA; Fig. 5b).

**Figure 6:**
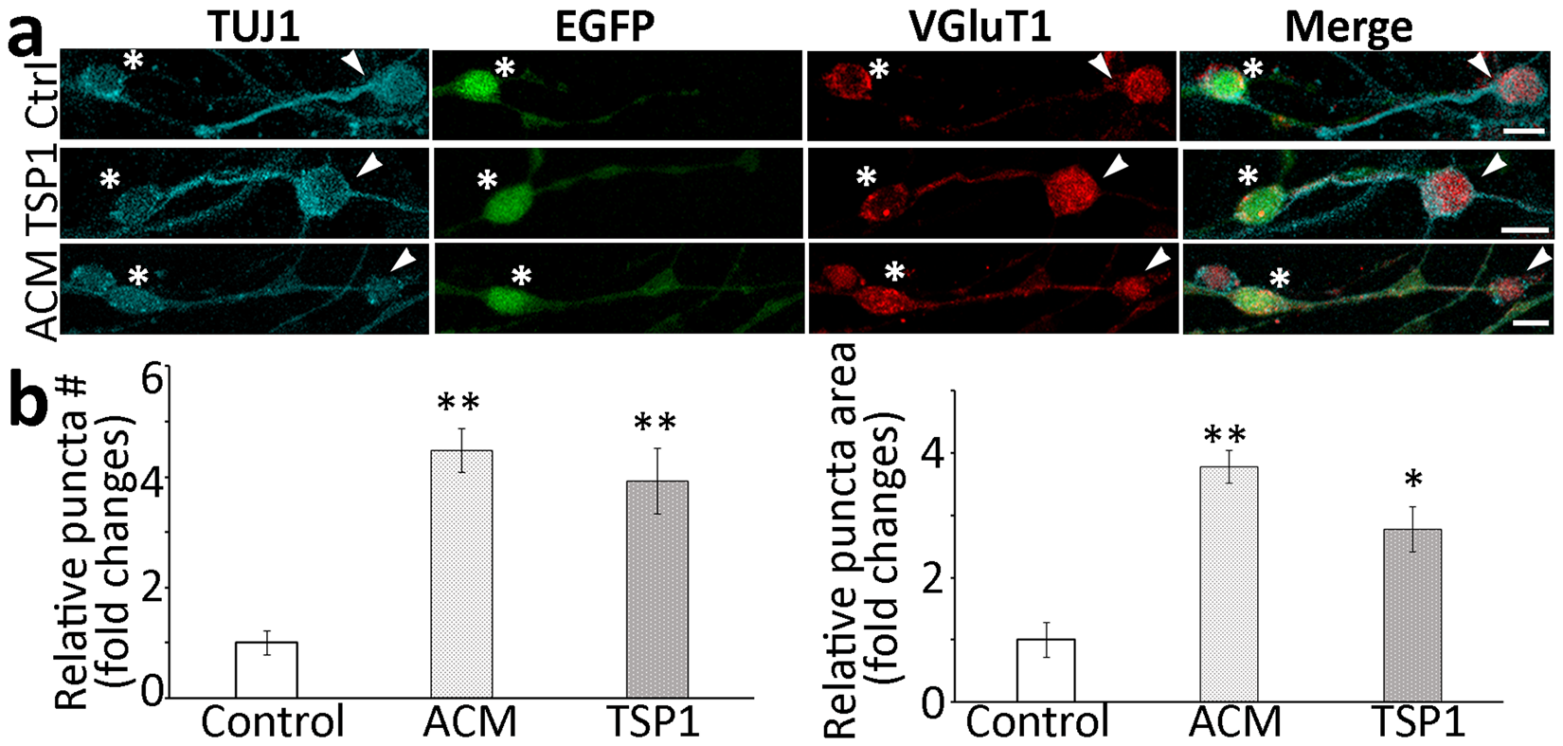
New connections between EGFP-ScNs and CN neurons expressed glutamatergic proteins. (**a**) TUJ1-expressing connections were seen between EGFP-ScNs (asterisks) and wild type CN neurons (arrowheads), which also expressed the glutamatergic marker VGluT1 in the control (Ctrl), TSP1, and ACM groups. (**b**) The quantitative study revealed that relative number and area of VGluT1 puncta along connections were lower in the control group, whereas they were significantly higher in the ACM and TSP1 groups (mean ± standard error shown in the figure; *indicates P < 0.05, **indicates P < 0.01, ANOVA followed by Tukey post hoc test). Scale bar: 10 µm in (**a**).

Since SGNs conduct auditory signals to the CN via the neurotransmitter glutamate, newly-formed synapse-like structures were tested by glutamatergic antibody immunostaining. It was found that new synapse-like formations were labeled by anti-VGluT1 antibodies (Fig. 6a), suggesting that these synapse-like structures were likely glutamatergic synapses. In a quantitative study, significantly more puncta were found along ScN-CN connections in the ACM and TSP1 groups (P < 0.01, ANOVA; Fig. 6b).

**Figure 7:**
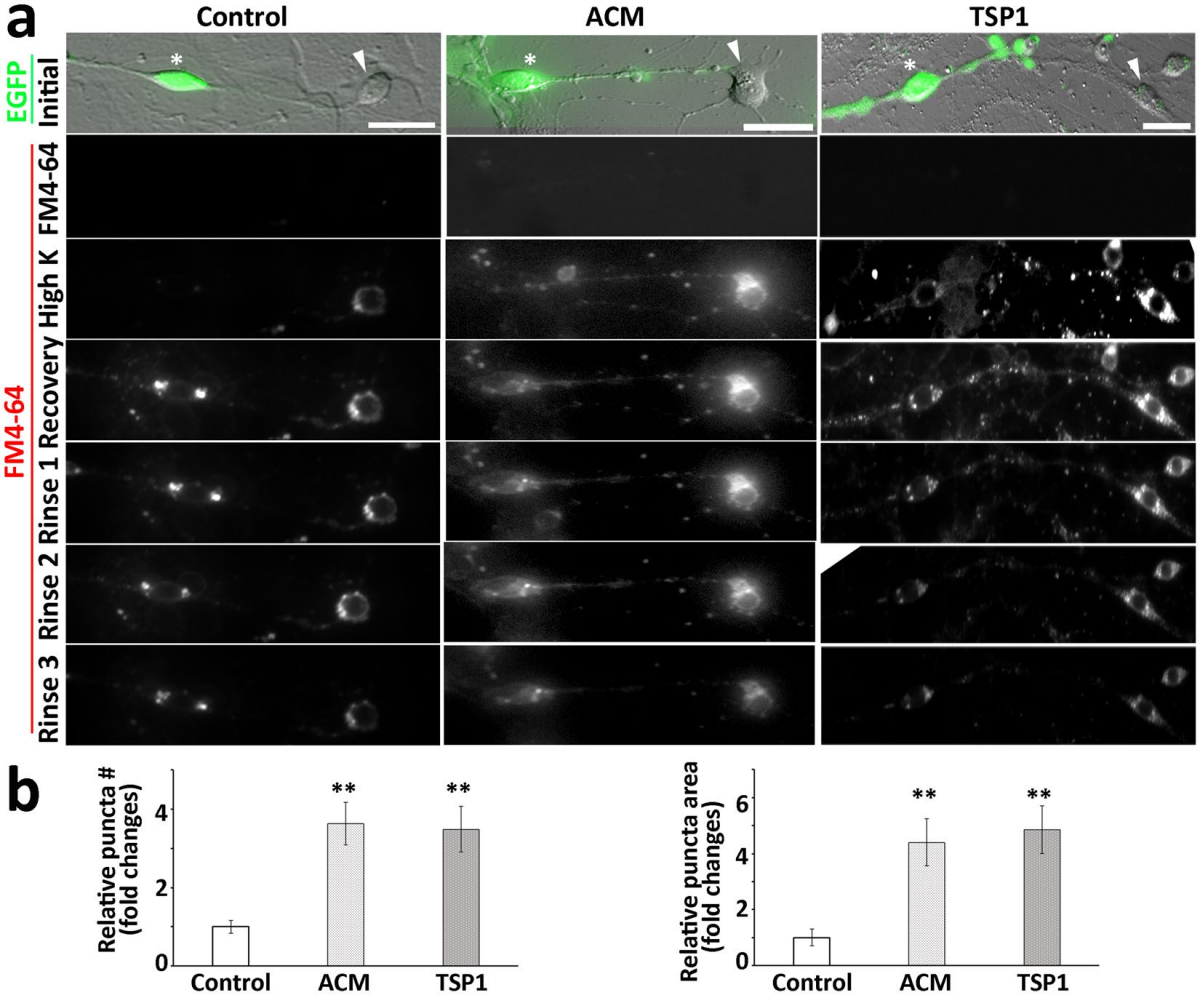
The synaptic vesicle recycling study of connections between EGFP-ScNs and CN neurons. (**a**) EGFP-ScNs and CN neurons were co-cultured for 4–6 days, followed by FM4-64 synaptic vesicle recycling study. In initial exposure to FM4-64 for 1 min, no obvious staining was found in 3 groups. After high potassium stimulation, obvious staining was found in the soma and neurite outgrowths in the ACM and TSP1 groups. In the recovery stage, significant vesicle staining was observed in soma and nerve terminals of ACM and TSP1 groups. During rinse stages, the staining of vesicles remained distinct in the soma and neurite outgrowths. (**b**) The puncta at the recovery stage was used for the quantitative study. It was observed that relative number and area of FM4-64 vesicle along connections were lower in the control group, whereas they were significantly higher in ACM and TSP1 groups (mean ± standard error shown in the figure; **indicates P < 0.01, ANOVA followed by Tukey post hoc test). Scale bar: 25 µm in (**a**).

A red fluorescence format styryl dye FM4-64 was utilized to perform a synaptic vesicle recycling study^18,19^. In initial exposure to the dye for 1 min, no obvious staining was found in the control, ACM, and TSP1 groups (Fig. 7a). The dyes were internalized (endocytosis) during synapse vesicle retrieval after high potassium stimulation, in which dye internalization was significantly increased and obvious staining was found in the soma and neurite outgrowths of the control, ACM, and TSP1 groups. In the recovery stage, significantly more FM4-64 stained vesicles were observed in soma and nerve terminals of ACM and TSP1 groups (P < 0.01, ANOVA; Fig. 7b). During the rinse stages, the vesicle staining of three groups became weaker, but remained distinct in the soma and neurite outgrowths at the end of experiment. This study revealed that synaptic vesicles along ScN-CN neuron connections were able to be stained by FM dye and transported to the nerve terminals, suggesting that new synapse-like formations may be functional.

## Discussion

In this study, we developed a novel stem cell-based co-culture system to understand whether mouse stem cell-derived PNS neurons were able to form synaptic connections with mouse CNS neurons. We found that inner ear NSC-derived ScNs formed synapse-like structures with CN neurons *in vitro*. ACM was able to stimulate synapse-like formation, in which TSP1 seemed to play an essential role. Newly-formed synapse-like structures expressed synaptic features including pre- and post-synaptic proteins. Synaptic vesicle recycling studies indicate that new synapse vesicles were functional.

SGNs transfer auditory signals from the peripheral auditory system to CN neurons of the CNS. Stem cell-based neuronal regeneration has been studied and it is known that SGN-like neurons are able to be generated both *in vitro* and *in vivo* ^5,6,10,11,20–22^. Chen *et al*. reported that transplantation of stem cell-derived neural progenitors led to hearing function improvement^5^. However, synapse formation between exogenous cells and native CN neurons has not been fully understood. A novel co-culture system has been developed in this report to address this issue. For stem cell-derived neurons, a recently reported SGN-NSC model was used to produce ScNs for the co-culture system. Because NSCs can be maintained for several passages, it provides a stable stem cell-derived neuronal resource for the co-culture system. Additionally, a novel CN neuron primary culture method was generated and optimized in this research. It was found that CN neurons were able to be maintained *in vitro* for at least 9–12 days with detection of cells expressing neuronal proteins in the presence of NGF. By utilizing the advantages of ScN and CN neurons, co-cultured neurons can be maintained in the culture dish for at least 9–12 days, which may be sufficient for studying synapse formation. Establishment of this novel co-culture system provides a useful *in vitro* system to study the morphology, gene and protein expression, as well as function of new synapse formation between stem cell-derived neurons and native neurons. Moreover, since the co-culture system can be observed daily, it may be utilized to test proteins and factors that are able to stimulate synapse formation. Additionally, the results of the *in vitro* co-culture study may be applicable to the *in vivo* system in future studies. In the meantime, this co-culture system has weaknesses that need to be improved in the future research. For instance, once the methods for maintaining the tonotopic structure of the CN tissue is identified *in vitro*, intact CN tissue culture may be used for co-cultures to study whether tonotopic connections are formed between ScNs and subtypes of CN neurons.

In the new co-culture system, synapse-like formations were observed between the same type of neurons including ScN-ScN and CN-CN connections, and notably ScN-CN connections, which were focused in this study. The formation of ScN-CN connections suggests that inner ear NSC-derived neurons were able to form neural contacts with native CN neurons. The underlying mechanism of ScN-CN synapse formation has not been fully understood. Glial cell-released proteins, factors, and/or cytokines may play important roles, as ACM seems to stimulate expression of synaptic vesicle SV2 along ScN-CN connections. Additionally, ScNs were derived from inner ear NSCs, which may have a default program to form synapses with CN neurons as during normal development, in which SGNs extend neurites to the CN^23–25^. These speculations deserve independent experiments in the future research.

ACM has been reported to stimulate retina ganglion neuron synaptogenesis^16,26^, maintain hippocampus neuron presynaptic plasticity^27^, and prevent synapse and spine abnormalities in the Fragile X syndrome mouse model^28^. In the stem cell research, a recent report suggests that ACM is able to stimulate synapse-like formation among ScNs^10^. In the current study, it is observed that ACM is able to stimulate synapse-like formation between ScNs and CN neurons, which suggests that ACM-exerted synaptogenic effects apply to two types of neurons. Interestingly, these two types of neurons are PNS neurons and CNS neurons, indicating that ACM exerts synaptogenic effects to connect PNS and CNS neurons.

It is noted that a few SV2 puncta were observed in the control group, in which ACM was not added to the co-culture, suggesting that other factors may be involved in stimulating SV2 expression in the control group. One possibility is the astrocytes of CN tissues. It is likely that CN astrocytes release proteins and/or cytokines to assist SV2 expression in the co-culture, which is similar to the supplemented ACM. However, GFAP-expressing cells are only 10.9 ± 4.2% (mean ± standard deviation) of the cell population in the serum-free co-culture. In contrast, there are 94.3 ± 4.2% (mean ± standard deviation) GFAP-expressing cells in the cortex astrocyte culture (serum-containing) used for ACM collection (supplemental Fig. 2). Thus, the ratio of GFAP-expressing cell in cortex astrocyte: co-culture is approximately 9:1. Additionally, cortex astrocyte ACM is concentrated at 50:1 during preparation. Though ACM is diluted 1:10 for co-culture, the overall ratio of astrocyte in ACM: co-culture is 45:1. Therefore, the pre-existing factors in the co-culture have minimal effects compared to concentrated ACM. Total elimination of CN astrocytes may be investigated to have a “clean” ACM negative control in future studies.

TSPs are a group of multimeric extracellular matrix glycoproteins, which consist of subgroups A (trimeric TSP1 and TSP2) and B (pentameric TSP3, TSP4, and TSP5)^17^. TSPs play a variety of roles in modulating cell behavior by engaging specific cell surface receptors, such as CD36, CD47, and α2δ1^17,29,30^. Applying TSP1 to rat retinal ganglion cell cultures resulted in a several-fold increase in the number of excitatory synapses^16^. In cultured rat hippocampal neurons, TSP1 increased the speed of synapse formation in young neurons^31^. In the animal study, TSP1 and TSP2 were necessary for synaptic and functional recovery after stroke^26^. TSP family proteins were found to promote synapse formation and neurite outgrowth of human umbilical tissue-derived cells^32^. In order to determine whether TSP1 plays synaptogenic roles in PNS neuron-CNS neuron co-cultures, TSP1 immunodepletion and TSP1 knockout mouse models were used in this study. It was found that ACM-induced synaptogenic effects were abolished in TSP1-IP-ACM and TSP1-KO-ACM groups, which was rescued when purified TSP1 protein was supplemented. Additionally, the receptor for TSP1 (α2δ1) was expressed on ScNs (supplemental Fig. 3). These studies suggest that TSP1 may play a fundamental role in ACM-induced synapse-like formation between stem cell-derived PNS neurons and native CNS neurons. Identifying the protein responsible for synapse formation in stem cell-based co-culture will provide critical cues for the design of future *in vitro* and *in vivo* studies.

Newly-formed ScN-CN synapse-like structures exhibited multiple synapse characteristics. For instance, new synapse-like structures expressed the synaptic vesicle protein SV2, the presynaptic protein synapsin, and the postsynaptic protein PSD93. Confocal-based co-localization test suggests that pre- and post-synapse proteins were co-localized. Further, new synapse-like structures showed the glutamatergic synapse protein VGluT1, which is a key feature of afferent SGNs. These results suggest that new ScN-CN connections expressed synapse proteins and thus they likely possess the features of synapse-like formations. In the functional tests using synaptic vesicle recycling study, ScNs and CN neurons were labeled by FM dye after high potassium stimulation, and stained vesicles moved to the nerve terminals during the recovery and rinse stages. The quantitative study shows that significantly more FM4-64 stained vesicles were found in ACM and TSP1 groups in the recovery stage. These results indicate that these new neural connections may possess functional synapse vesicles.

In summary, a novel stem cell-based co-culture system has been established in this study. Stem cell-derived PNS neurons are proved to form synapses with the native auditory CNS neurons *in vitro*. ACM and TSP1 were capable of stimulating synapse formation between PNS and CNS neurons in co-cultures. These results reveal the possibility of utilizing stem cell-based cell therapy to replace damaged SGNs and rebuild the auditory circuit from the inner ear to the central auditory system. These findings may be applicable to other neural system, in which peripherally regenerated PNS neurons may be reconnected to native CNS neurons to treat various types of neurological degeneration and/or disorders, which involve damages to PNS neurons and injuries to neural contacts connecting PNS and CNS neurons.

## Methods

### CN tissue primary culture

The care and use of the animals have been approved by local Institutional Animal Care and Use Committee. Methods were carried out in accordance with the approved guidelines. Postnatal day 3–5 Swiss Webster mice were decapitated, and heads were transferred to the dish containing ice cold DMEM/High Glucose (Hyclone). Cerebellum and mesencephalon were removed to expose brainstem, and the cochlear nucleus (CN) that was located on the dorsolateral surface of the brainstem was removed and transferred to 0.025% Trypsin (Invitrogen, diluted in HBSS) at 37 °C for 10–12 min, followed by mechanical dissociation using polished glass pipet. Dissociated CN tissues were transferred to culture wells containing 45% DMEM/F12, 45% neurobasal medium, 10% fetal bovine serum (FBS), 55 nM 2-mercaptoethanol, 0.1% penicillin/streptomycin (all from Invitrogen), and maintained in 5% CO_2_ incubator at 37 °C. Cells were observed daily using phase contrast microscopy or differential interference contrast (DIC) microscopy, and half of the culture medium was replaced every 2–3 days.

To support CN neuron survival, 0, 1, 5, 10, and 20 ng/ml nerve growth factor (NGF, Invitrogen) was added to the culture medium for 5 days (n = 6 samples in each group), followed by immunofluorescence using anti-class III β-Tubulin (TUJ1, 1:500; Aves) and anti-NeuN (1:200; Millipore) antibodies (refer to the Immunofluorescence section). TUJ1 positive, NeuN positive, and double-labeled cells were observed by Leica SPE confocal or epifluorescence microscopy, counted using ImageJ with the cell counter plugin (NIH), and shown in mean ± standard deviation. Analysis of variance (ANOVA) with Tukey post hoc tests were used for statistical analysis. P < 0.05 was considered statistically significant in this study.

To determine neural cell types in the CN primary culture, dissociated CN tissues were cultured for 3–12 days in the presence of NGF (n = 6 samples in each group), and fixed in 4% paraformaldehyde, followed by immunofluorescence using anti-TUJ1, anti-glial fibrillary acidic protein (GFAP 1:200; Santa Cruz Biotechnology), and anti-myelin oligodendrocyte glycoprotein (MOG 1:500; Millipore) antibodies (refer to the Immunofluorescence section). The samples were observed and imaged by Leica SPE confocal or Leica epifluorescence microscopy. Cells were counted as above and analyzed by ANOVA with Tukey post hoc test.

To study cell proliferation, EdU (200 ng/ml, Invitrogen) was added to the CN culture medium at culture day 1, 4, 7, and 10 for 48 hr, and CN tissues were fixed at culture day 3, 6, 9, and 12 (n = 6 samples per group)^10^. EdU incorporation was studied according to the manufacturer’s instructions (Invitrogen), followed by immunofluorescence using anti-TUJ1 and anti-GFAP antibodies. Cells were counted as above and analyzed by ANOVA with Tukey post hoc test.

### ScN generation and ScN-CN co-culture

ScN primary culture was performed as previously described^10^. Postnatal day 1–3 EGFP transgenic mice (The Jackson Laboratory) were used for SGN tissue collection and neural sphere generation. After EGFP-SGN-derived neural spheres were passaged 3 times (2–3 weeks), they were dissociated by TryplE (Invitrogen) and plated into culture wells containing DMEM/F12, 10% FBS, and 20 ng/ml BDNF (R&D system) for ScN production. Cells were maintained for 6–12 days, followed by immunofluorescence using anti-TUJ1, anti-Gata3 (1:200; Sigma), and anti-VGluT1 (1:200; Invitrogen) antibodies.

For ScN-CN co-culture, CN tissues were dissected, dissociated and seeded to culture wells overnight as above. EGFP-SGN-derived neural spheres were dissociated with TryplE, followed by seeding into culture wells containing dissociated CN tissues. The medium for co-culture was 50% DMEM/F12, 48% neurobasal medium, 1% N2, 1% L-glutamine, 55 nM 2-mercaptoethanol, 0.1% penicillin/streptomycin, 20 ng/ml NGF, and 20 ng/ml BDNF. Co-culture samples were maintained in the incubator as above and observed daily with Leica light and epifluorescence microscopy.

### Astrocyte conditioned medium (ACM) and thrombospondin-1 (TSP1) treatment

Postnatal day 0–1 wild type Swiss Webster and TSP1 knockout (B6.129S2-Thbs1tm1Hyn/J, The Jackson Laboratory) mice were used for wild type ACM and TSP1-knockout ACM (TSP1-KO-ACM) collection respectively. ACM was collected and prepared as described previously^10^, and added to co-cultures at the final dilution of 1:10. Briefly, cortex tissues were isolated, dissociated by pipetting, and cultured in 90% DMEM High glucose, 10% FBS, and 0.1% penicillin/streptomycin (all from Hyclone). When cells reached 60–70% confluence, they were passaged using TryplE. At the first passage, a small amount of cells were seeded on coverslips for GFAP immunostaining (supplemental Fig. 2). From passage 2 (∼2 weeks after primary culture), the conditioned medium was collected for 4–5 passages and filtered with a 0.45 μm filter (Millipore) to remove cell debris. ACM was concentrated with 30 kDa molecular weight cutoff tubes (Millipore) using the manufacturer’s protocol (50:1 concentration). Concentrated ACM was filtered with 0.22 μm syringe filters (Millipore), protein concentration measured using Bradford assay (Bio-Rad), and stored at −80 °C.

Half of the culture medium was replaced every 2–3 days. Co-cultures were observed daily by epifluorescence microscopy for 4–6 days to observe connections between neuronal-like cells, followed by fixation and immunofluorescence using anti-TUJ1, anti-synaptic vesicles (SV2,1:100; Developmental Studies Hybridoma Bank, DSHB), anti-synapsin (1:100; Santa Cruz), anti-PSD93 (1:200; Neuromab), and/or anti-VGluT1 (1:200; Synaptic Systems) antibodies. Samples were observed using Leica SPE confocal or epifluorescence microscopy. The Pearson’s R test was used for analyzing synapsin and PSD93 co-localization by the Leica LAS AF Lite Co-localization software.

For immunodepletion, Dynabeads (Invitrogen) were incubated with anti-TSP1 antibody (25 μg/ml, R&D system) or mouse IgG whole protein (25 μg/ml, Jackson ImmunoResearch; the mock control) at room temperature for 30 min. Following rinse, 200 μl of treated Dynabeads conjugated with either anti-TSP1-antibodies or mouse IgG were incubated with 500 μl ACM and rotated at room temperature for 1 hr. Beads were removed by magnet, and TSP1-immunodepleted ACM (TSP1-ID-ACM) and the mock control (mock-ID-ACM) were collected and stored at −80 °C for further use.

To test the optimal TSP1 concentration, 0, 2, 5, 10, 20 nM purified TSP1 proteins (R&D systems) were added to the co-culture medium, and half of the medium was replaced every 2–3 days. Co-cultures were observed and fixed for immunofluorescence and analysis as above.

SV2 puncta was analyzed using previously reported methods^10^. Briefly, to quantitatively compare SV2 puncta, the number of the control group was established as 1. The SV2 numbers of the other groups were divided by the control group to obtain relative SV2 numbers. The number and area of SV2 puncta along EGFP-ScN and CN neuron connections, which was determined as 0.1–3 µm^2^ in size, were quantified by ImageJ using the particle analyze feature with color threshold (n = 6–7 samples per group, from 3 independent experiments, 2–3 samples per experiment). In each sample, the number and area of SV2 puncta along ScN and CN connections were calculated and presented as mean ± standard error. Similarly, the number and area of puncta in of the pre-/post-synaptic, VGluT1, and recovery stage of the FM4-64 study were analyzed (n = 6 samples per group). Normality test was performed by SPSS, and either Mann-Whitney U test or ANOVA (followed by Tukey post hoc test) was applied for analysis. P < 0.05 was considered statistically significant.

### Immunofluorescence and western blotting

After fixation with 4% paraformaldehyde, cell samples were treated with PBS containing 5% donkey serum and 0.2% Triton-X100 for 20–30 min at room temperature. Samples were incubated in the primary antibody at 4 °C overnight, followed by corresponding secondary antibody incubation at room temperature for 2 hr. Secondary antibodies included AMCA, Alexa Fluor 488, Cy3, and Alexa Fluor 647 conjugated donkey anti-mouse, goat, rabbit, or chicken antibodies (Jackson Immunoresearch). 4,6-Diamidino-2-Phenylindole (DAPI; Invitrogen) was used to label all nuclei. Samples were observed and imaged by Leica 3000B epifluorescence microscopy and/or Leica SPE confocal microscopy.

For western blotting, purified TSP1 protein, ACM collected from wild type Swiss Webster mice, TSP1-ID-ACM, mock-ID-ACM, TSP1-KO-ACM, and DMEM/F12 were loaded to blotting gels. Antibodies used in western blotting included goat anti-TSP1 antibodies, donkey anti-goat HRP-conjugated antibodies, and HRP standard protein (all from Bio-Rad). SuperSignal West Femto Stable Peroxide Solution and SuperSignal West Femto Luminol/Enhancer Solution (ECL, all from Thermo scientific) were applied to blotting membrane for protein detection. Images were captured using a ChemiDoc-It 2 imaging system (UVP).

### FM4-64 synaptic vesicle recycling study

Culture day 4–6 co-culture samples were used for FM4-64 synaptic vesicle recycling assay^18,19^. FM4-64 (Invitrogen) was diluted to 10 µM in the Tyrode’s solution (in mM: 125 NaCl, 2 KCl, 2 MgCl_2_, 30 D-glucose, and 25 HEPES; all from Sigma). Co-culture samples were incubated in FM4-64 solution for 1 min, and treated by the high potassium solution (in mM: 59 NaCl, 70 KCl, 1 MgCl_2_, 30 D-glucose, 25 HEPES, and 2 CaCl_2_; all from Sigma) in the presence of the dye for 90 seconds, followed by recovery in the FM4-64 solution for 15 min. The dye was washed off with ice cold HBSS (Mg^2+^ and Ca^2+^ free) every 3 min for 15–18 min. The samples were observed with a 40x objective by Leica DMi8 epifluorescence microscopy.

### Data availability statement

All data generated or analysed during this study are included in this published article (and its Supplementary Information files).

## Electronic supplementary material

Supplemental info
