# Supplementary material for: Stimulation of synapse formation between stem cell-derived neurons and native brainstem auditory neurons
Source: Sci Rep. 2017 Oct 23;7:13843. doi: 10.1038/s41598-017-13764-8 (PMC5653851; doi:10.1038/s41598-017-13764-8)
Supplement: Supplementary file 1 — Supplemental info [file 41598_2017_13764_MOESM1_ESM.pdf]

Stimulation of synapse formation between stem cell-derived neurons and native brainstem auditory neurons

Zhengqing Hu, Zhenjie Liu, Xiaoyang Li, and Xin Deng

Original western

blotting gel image

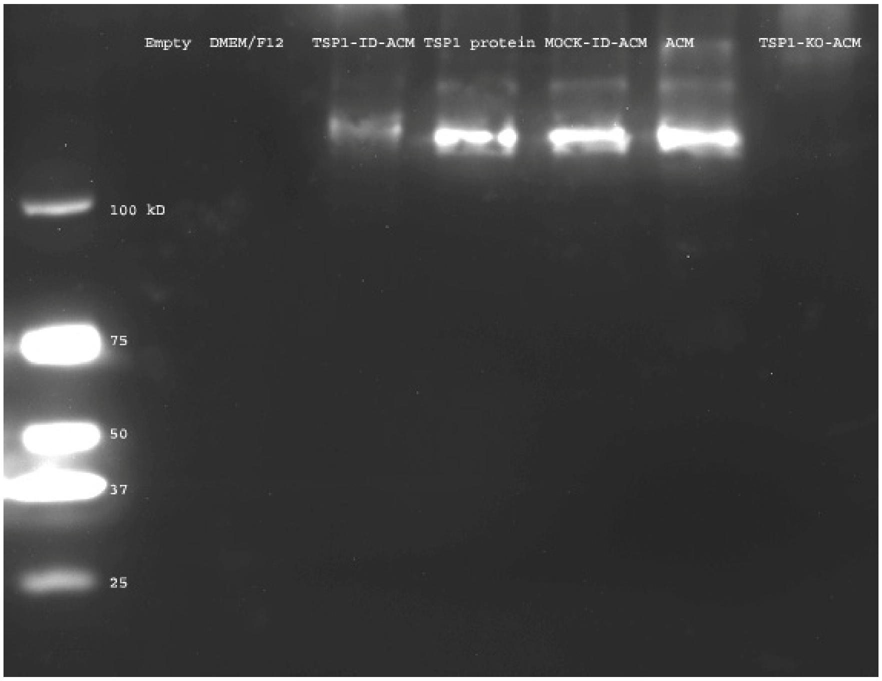

Black-white

Inverted image

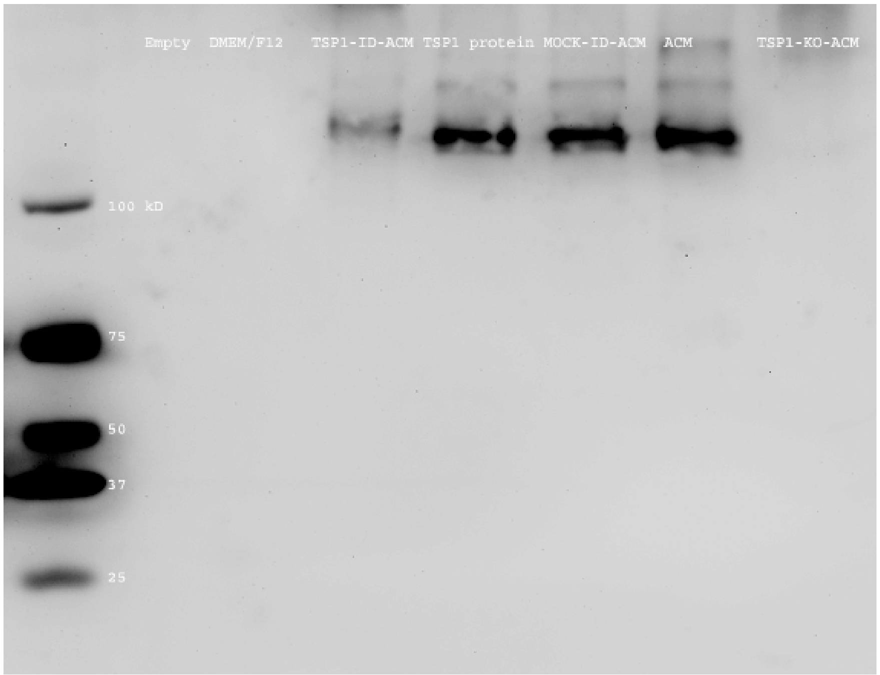

Supplemental figure 1. Original and black-white inverted images of western blotting of Figure 4a.

Western blotting gels showed clear TSP1 bands in TSP1, mock, and ACM groups, a weak band in TSP1 immunodepleted ACM group (TSP1-ID-ACM), and no bands in empty, DMEM/F12, or TSP1 knockout ACM (TSP1-KO-ACM) groups.

The ACM band represents concentrated conditioned medium collected from wild type astrocyte culture. TSP1-ID indicates that TSP1 was immunodepleted from wild type ACM using TSP1 specific antibodies. TSP1-KO stands for concentrated conditioned medium collected from TSP1-knockout mouse astrocyte culture. The study also included pure TSP1 protein, culture medium DMEM/F12, and an empty line (without adding any protein).

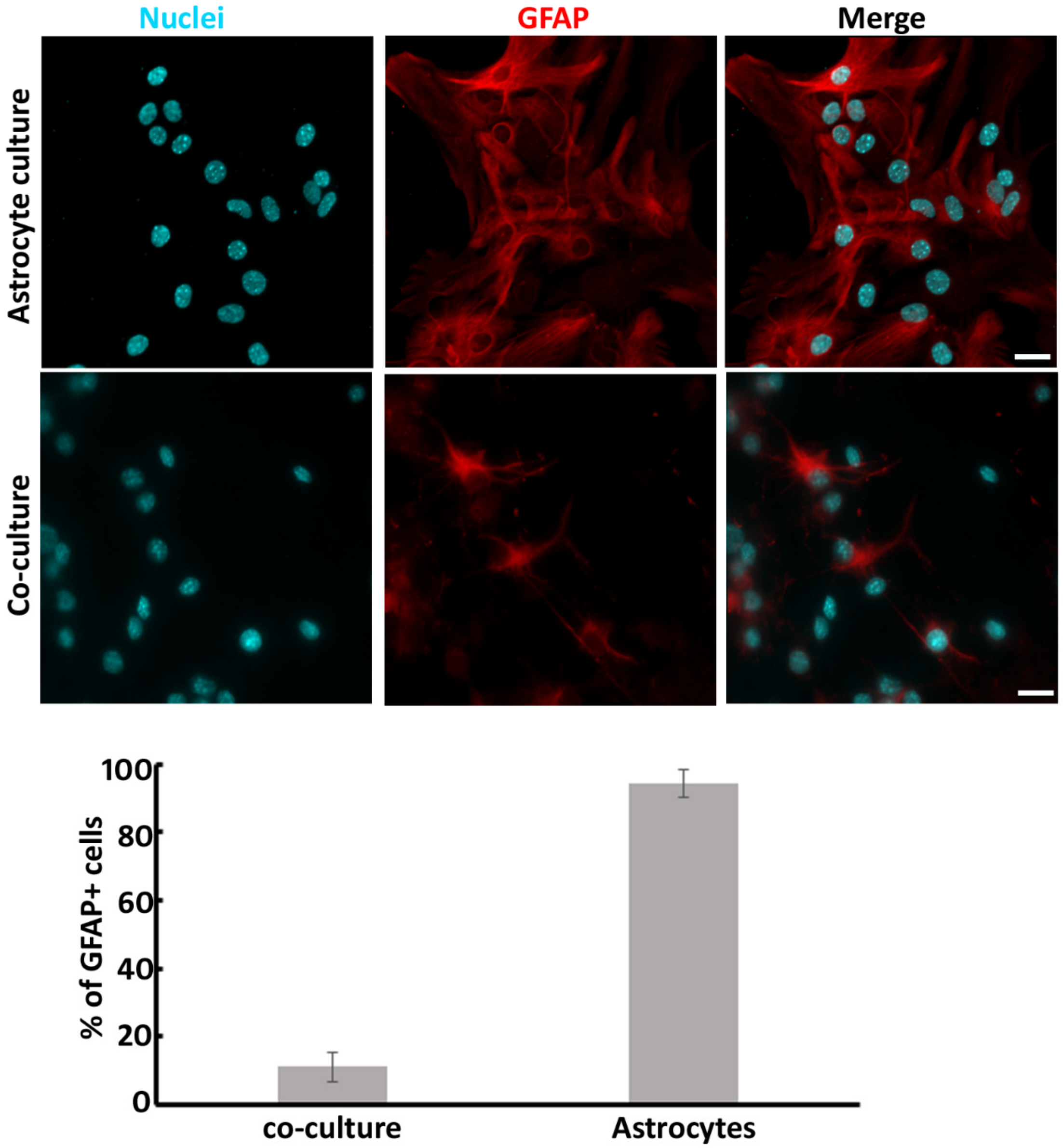

Supplemental figure 2. Percentage of GFAP+ cells in the astrocyte culture and co-culture.

A number of cells were GFAP+ in the astrocyte culture for ACM collection, whereas very few GFAP+ cells were found in the co-culture, which was statistically significant ($P<0.01$; Student's *t*-test, two tails, two-sample assuming unequal variances).

Scale bar: 25 µm.

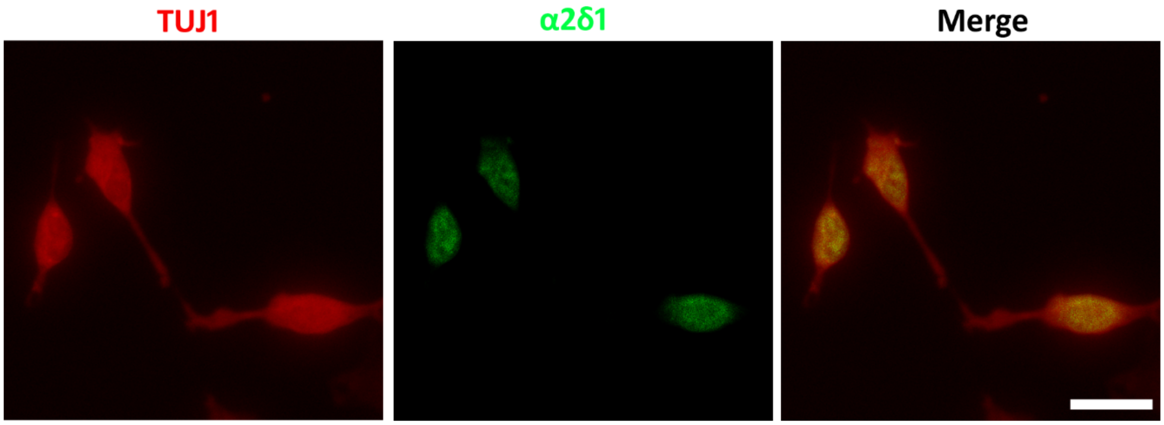

Supplemental figure 3. α2δ1 expression on ScNs.

α2δ1 immunostaining suggests that expression of α2δ1 was detected on ScNs.

Scale bar: 25 μm.
